# Hepatic Oxidative Stress in Fructose-Induced Fatty Liver Is Not Caused by Sulfur Amino Acid Insufficiency

**DOI:** 10.3390/nu3110987

**Published:** 2011-11-18

**Authors:** Sachin S. Kunde, James R. Roede, Miriam B. Vos, Michael L. Orr, Young-Mi Go, Youngja Park, Thomas R. Ziegler, Dean P. Jones

**Affiliations:** 1 Division of Gastroenterology, Hepatology and Nutrition, Emory Children’s Center, 2015 Uppergate Dr NE, Emory University School of Medicine, Atlanta, GA 30322, USA; Email: mvos@emory.edu; 2 Whitehead Biomedical Research Bldg. Division of Pulmonary, Allergy and Critical Care Medicine, 615 Michael St, Emory University School of Medicine, Ste 205P, Atlanta, GA 30322, USA; Email: jroede@emory.edu (J.R.R.); mlorr@emory.edu (M.L.O.); ygo@emory.edu (Y.-M.G.); youngja.park@emory.edu (Y.P.); youngja.park@emory.edu (D.P.J.); 3 Department of Medicine, GG23 Emory University Hospital, 1600/002/1AA, 1364 Clifton Rd, Atlanta, GA 30322, USA; Email: tzieg01@emory.edu

**Keywords:** cystine, methionine, thioredoxin, redox potential, mitochondria, obesity

## Abstract

Fructose-sweetened liquid consumption is associated with fatty liver and oxidative stress. In rodent models of fructose-mediated fatty liver, protein consumption is decreased. Additionally, decreased sulfur amino acid intake is known to cause oxidative stress. Studies were designed to test whether oxidative stress in fructose-sweetened liquid-induced fatty liver is caused by decreased *ad libitum* solid food intake with associated inadequate sulfur amino acid intake. C57BL6 mice were grouped as: control (*ad libitum* water), fructose (*ad libitum* 30% fructose-sweetened liquid), glucose (*ad libitum* 30% glucose-sweetened water) and pair-fed (*ad libitum* water and sulfur amino acid intake same as the fructose group). Hepatic and plasma thiol-disulfide antioxidant status were analyzed after five weeks. Fructose- and glucose-fed mice developed fatty liver. The mitochondrial antioxidant protein, thioredoxin-2, displayed decreased abundance in the liver of fructose and glucose-fed mice compared to controls. Glutathione/glutathione disulfide redox potential (E_h_GSSG) and abundance of the cytoplasmic antioxidant protein, peroxiredoxin-2, were similar among groups. We conclude that both fructose and glucose-sweetened liquid consumption results in fatty liver and upregulated thioredoxin-2 expression, consistent with mitochondrial oxidative stress; however, inadequate sulfur amino acid intake was not the cause of this oxidative stress.

## 1. Introduction

Consumption of refined sugars has increased over the past decades [1,2] and fructose, in particular, has been implicated as a contributing factor in the development of metabolic diseases [3]. These diseases include conditions such as obesity, dyslipidemia, insulin resistance, diabetes, high blood pressure [4,5,6,7,8,9,10,11,12,13], and non-alcoholic fatty liver disease [14,15,16,17,18]. The etiology of fructose-mediated metabolic disturbances is not completely understood; however, it is likely due to a multifactorial disease process [4,5,9,19,20]. Fructose consumption and its metabolic alterations, including fatty liver, have been associated with oxidative stress [21,22,23,24,25,26,27,28,29,30,31,32,33], which may play role in the pathogenesis of these conditions.

In animal models, fructose-sweetened beverage consumption is associated with decreased food and protein intake [34,35,36]. Therefore, inadequate ingestion of macronutrients and energy from solid foods may be important in the metabolic alterations attributed to fructose-sweetened beverages [20,37,38,39,40,41,42,43]. In rodents, when fructose-sweetened liquid is provided *ad libitum*, liquid intake increases and solid food consumption decreases, while total energy intake is maintained at a level comparable to controls [34,44,45]. This resultant decrease in solid food results in decreased protein consumption and insufficient intake of non-essential and essential amino acids, which includes the sulfur amino acids, *i.e.*, cysteine (Cys) and methionine. 

All cells have thiol-dependent antioxidant systems that are critical in redox regulation of cellular processes, like gene transcription, and protection against oxidative stress [46]. Major thiol-containing redox couples (GSH/GSSG, thioredoxin (Trx) and peroxiredoxin (Prx)) contain Cys as a central thiol and oxidation of these residues provide useful markers of oxidative stress [47]. In humans, Cys deficiency can lead to plasma oxidative stress [48], and in rodents, dietary sulfur amino acid insufficiency causes plasma and tissue level oxidative stress [49,50], as shown by steady-state redox potentials (E_h_) for GSH/GSSG and Cys/CySS redox couples. Because fructose-sweetened beverages decrease dietary sulfur amino acid intake and sulfur amino acid insufficiency causes oxidative stress, it is possible that decreased sulfur amino acid intake may contribute to oxidative stress observed in fructose-induced fatty liver. Therefore, the purpose of this study was to determine if oxidative stress associated with fructose-induced fatty liver was due to decreased sulfur amino acid intake. In the experimental design, pair-feeding of mice with the amount of solid food consumed by the fructose group resulted in substantial energy deficiency in the pair-fed group. Despite this difference in energy intake, the pair-fed group did not show hepatic oxidative stress either in terms of thiol/disulphide levels or mitochondrial thioredoxin-2 or cytoplasmic/nuclear peroredoxin-2 levels. Thus, the results show that fructose- or glucose-induced fatty liver is not an artefact due to oxidative stress caused by insufficient protein intake. 

## 2. Materials and Methods

### 2.1. Animals and Feeding Protocol

This protocol was approved by the Institutional Animal Care and Use Committee at Emory University and performed according to NIH guidelines. The study outlined below was repeated in two separate experiments to confirm findings. Results were similar for both experiments and combined for presentation. 

Five week-old C57BL6 male mice (*N* = 39) were purchased from Charles River Laboratory. Anhydrous dextrose and D-fructose were purchased from Harlan Teklad. Mice were housed in individual cages in a pathogen-free barrier facility. During an initial acclimatization period of 1 week, all mice received water *ad libitum* and solid food *ad libitum* (LabDiet-5001, AIN 93M, PMI Nutrition International). Methionine (Met) and Cys content of the diet were 0.67% and 0.31%, respectively. At 6 weeks of age, mice were allocated to four groups: control (*ad libitum* water and *ad libitum* solid food; *n* = 10), fructose (*ad libitum* 30% fructose-sweetened water (w/v) and *ad libitum* solid food; *n* = 11), glucose (*ad libitum* 30% dextrose-sweetened water (w/v) and *ad libitum* solid food; *n* = 10) and a pair fed group (*ad libitum* water, pair fed to fructose for solid food intake of F; *n* = 8). The pair-fed animals were started on dietary intervention one day after starting the fructose-fed mice and the mean daily individual food consumption of the fructose group was fed to individual pair-fed mice. Mice were examined daily for signs of dehydration and distress. Body weight and water and food consumption, were measured every other day. After 5 weeks, at sacrifice, animals were anesthetized with 80 mg/kg ketamine and 6 mg/kg xylazine i.p. Blood was collected from a cheek and 50 µL of plasma was processed for Cys, CySS, GSH and GSSG analyses by high performance liquid chromatography (HPLC) as previously outlined [49,50]. Portions of liver (approximately 5 mg) were immediately added to preservation solution for redox measurements [49,50]. Additional samples were snap frozen in liquid nitrogen or placed in OCT compound for cryosectioning and Oil Red O staining and stored at −80 °C. Because of a laboratory error, samples for Oil Red O staining were not available for the glucose-treated group. Because glucose-induced fatty liver is established in the literature [29,51] and confirmed by the triglyceride measurements obtained in the present studies, the experiment was not repeated to obtain this additional control.

### 2.2. Analytical Methods

Plasma and hepatic Cys, CySS, GSH and GSSG concentrations were measured using HPLC. Methods were adopted from Jones *et al.* and Nkabyo *et al.* [49,52,53]. Protein concentration of liver samples was measured using the Bradford assay. E_h_Cyss and E_h_GSSG were calculated using the Nernst Equation. Percent of oxidized thiol (% CySS and % GSSG) was calculated with respect to thiol concentration (oxidized + reduced + mixed disulfide Cys-GSH). Plasma Met concentration was measured as an additional indicator of plasma sulfur amino acid levels using liquid chromatography mass spectrometry (LC-FTMS) with electrospray ionization in positive ion mode essentially as described by Johnson *et al.* [54]. Plasma samples with visible hemolysis were excluded. Plasma extracts were separated using anion exchange chromatography and detected using LTQ-FT mass spectrometer (Thermo Fisher Scientific, San Jose, CA). Met (*m/z* 149.0510) was quantified relative to a stable isotopic internal standard. Hepatic triglycerides (TG) were assayed using a TG quantification kit (BioVision, CA; cat# K622-100). Liver samples (approximately 100 mg) were collected into 1 mL of 5× Triton-X solution, homogenized and processed per manufacturer’s instructions. For TG quantification, absorbance at 570 nm was calibrated to reference standards and data were expressed as nmol/100 mg of liver tissue. 

### 2.3. Western Blot Analyses

Hepatic protein abundance of thioredoxin-2 (Trx2) and peroxiredoxin-2 (Prx2) was analysed by Western blot. Liver (approximately 5 mg) was washed with ice cold PBS and immediately placed in 300 µL lysis buffer (1% NP-40, 0.5% Triton X-100, 50 mM Tris, 2 mM EDTA, 500 mM NaCl, protease inhibitor; pH 7.6), sonicated, vortexed, and centrifuged. Supernatant was used for protein quantification and Western blot analysis.

For all Western blots, 20 µg of protein was loaded in each well, separated using 15% SDS polyacrylamide gel electrophoresis and transferred to nitrocellulose membranes. After blocking, the membranes were incubated with primary antibodies specific to Trx-2 [55], Prx2 (Abcam16748), COX-IV (Abcam16056 as a mitochondrial control protein) or β-actin (Sigma-Aldrich A5441) for 1 h followed by the secondary antibody (IRDyeTM-800-conjugated anti-rabbit or anti-mouse antibody (Rockland Immuno chemicals, Gilbertsville, PA)) for 1 h. Protein bands were visualized by the Odyssey system (LiCor, Lincoln, NE) and quantified by densitometry.

### 2.4. Thiobarbituric Acid Reactive Substances Assay

A small amount of frozen liver tissue was placed in a 1.5 mL tube containing 200 μL of RIPA buffer. The samples were then sonicated for approximately 10 s to homogenize the tissue. Insoluble debris was pelleted via centrifugation and 100 μL of supernantant was added to 200 μL of 10% trichloroacetic acid. The samples were incubated on ice for 15 min and the precipitated protein was pelleted by centrifugation. 200 μL of supernatant was added to 200 μL of 0.67% (w/v) 2-thiobarbituric acid and incubated for 15 min at 100 °C. Samples were cooled to room temperature and absorbance at 532 nm was read on a microplate reader. 

### 2.5. Oil Red O Staining

Liver tissue preserved in OCT compound was cryosectioned into 10 µM sections, mounted onto microscope slides and stored at −10 °C until staining. Slides were allowed to acclimate to room temperature for approximately 10-15 min prior to staining. Tissue was then fixed in 10% neutral buffered formalin for 5 min and briefly washed in water. The slides were then rinsed in 60% isopropanol and placed into freshly prepared Oil Red O working solution. Slides were allowed to stain for 15 min at room temperature. After staining, slides were rinsed with 60% isopropanol and the nuclei were lightly stained with hematoxylin stain (approx. 5 dips). Lastly, the slides were washed 3 times in water and coverslips were applied using aqueous mounting media. The slides were then visualized on a light microscope at 100× magnification.

### 2.6. Statistical Analysis

Data are expressed as mean ± SEM. Statistical analysis was performed using SPSS 16.0 (IBM, Chicago, IL). ANOVA with Tukey’s post-hoc test was used to compare the groups. *p* ≤ 0.05 was considered statistically significant.

## 3. Results

### 3.1. Fatty Liver

**Figure 1:**
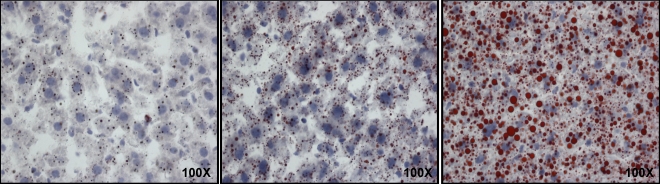
Oil Red O staining of frozen liver sections from control, pair-fed and fructose-fed mice. These sections are visualized at 100× magnification. The red staining corresponds to lipids and the blue stain corresponds to nuclei, respectively.

Fructose and glucose fed mice developed fatty liver as evidenced by significantly higher hepatic TG content (75 ± 4 nmol/100 mg and 70 ± 5 nmol/100 mg, respectively) compared to control and pair-fed mice (44 ± 1 nmol/100 mg and 41 ± 2 nmol/100 mg), respectively (each *p* < 0.05). To confirm the development of hepatic steatosis, frozen liver sections were stained with Oil Red O to visualize lipid accumulation (Figure 1). Results from fructose-fed mice confirmed fatty liver; samples from glucose-treated mice were not available, but glucose-induced fatty liver has been previously established [29,51] and confirmed in the present study by the TG measurements. Together, these data show that both fructose and glucose cause fatty liver.

### 3.2. Animal Characteristics

Body weight and food consumption data are detailed in Table 1. 

Initial body weights were similar for all groups. At the end of dietary treatment, the fructose and glucose-fed groups had a 10% and 25% weight gain respectively, compared to control. The pair-fed group lost weight during the course of the study because the design resulted in a relatively severe calorie restriction. However, these mice remained active and did not show any signs of distress. Consumption of fructose or glucose supplemented water resulted in a 38% and 54% decrease in solid food (and thus sulfur amino acid intake) consumption respectively, compared to control. Dietary liquid consumption was different across all the groups. Fructose-fed and glucose-fed mice had 42% and 98% higher, while the pair-fed mice had 56% lower liquid intake respectively compared to controls. Total energy intake (solid food + liquid) in fructose-fed (18 ± 1 kcal/day) and glucose-fed (19 ± 1 kcal/day) mice was similar to control mice (16 ± 1 kcal/day) (Table 1). As liquid consumption in the fructose and glucose groups increased, solid food intake decreased and total energy intake was similar compared to controls. Glucose-fed mice consumed about 30% more energy (11.4 ± 1 kcal/day) from liquid than fructose-fed mice (8.1 ± 1 kcal/day). Energy intake for mice in the pair-fed group (10 ± 0 kcal/day) was 52-62% of other groups.

**Table 1:** Animal weights and dietary intake ^#^.

Parameters	Control	Fructose	Glucose	Pair fed	*p*^†^
	*n* = 10	*n* = 11	*n* = 10	*n* = 8	
Initial weight (g)	20.1 ± 0.1 ^a^	20 ± 0.3 ^a^	19.9 ± 0.3 ^a^	19.6 ± 0.3 ^a^	0.7
Final weight (g)	25.5 ± 0.3 ^a^	28.1 ± 0.6 ^b^	31.8 ± 0.6 ^c^	17.1 ± 0.2 ^d^	<0.001
Solid food intake (gm/day)	3.9 ± 0.2 ^a^	2.4 ± 0.3 ^b^	1.8 ± 0.2 ^b^	2.4 ± 0 ^b^	<0.001
Liquid intake (mL/day)	4.8 ± 0.3 ^a^	6.8 ± 0.2 ^b^	9.5 ± 0.2 ^c^	2.1 ± 0.1 ^d^	<0.001
Cysteine intake (mg/day)	12.2 ± 0.7 ^a^	7.5 ± 0.9 ^b^	5.7 ± 0.5 ^b^	7.5 ± 0 ^b^	<0.001
Methionine intake (mg/day)	26.4 ± 1.5 ^a^	16.3 ± 20 ^b^	12.2 ± 1.2 ^b^	16.3 ± 0 ^b^	<0.001
Total caloric intake (kcal/day)	16.0 ± 1.0 ^a^	18.0 ± 1.0 ^a^	19.0 ± 1.0 ^a^	10.0 ± 0 ^b^	<0.001

^a,b,c,d^ Different superscripted letters indicate statistically significant (*p* < 0.05) difference between groups; ^#^ Data expressed as mean ± SEM; ^†^ ANOVA.

### 3.3. Plasma Sulfur Amino Acids

**Figure 2:**
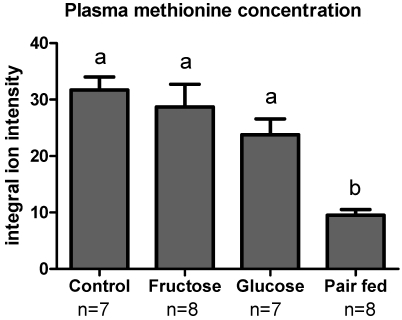
Plasma methionine concentration as measured by LC-FTMS. The data is expressed as integral ion intensity relative to internal standard. Pair-fed mice had significantly decreasedplasma methionine concentration compared to other groups. Different superscripted letters (a,b) indicate statistically significant differences among respective groups.

Plasma methionine levels were measured using LC-FTMS. The ion intensity of methionine in both fructose-fed and glucose-fed mice was similar to controls even though sulfur amino acid intake was lower (Figure 2). Pair-fed animals displayed methionine levels that were significantly lower than the other groups. Additionally, plasma Cys (Table 2) showed a trend similar to methionine and was also lower in pair-fed animals compared to other groups.

**Table 2:** High Performance Liquid Chromatography data of plasma and liver thiols ^#^.

Antioxidant thiols	Control	Fructose	Glucose	Pair fed	*p*^†^
	*n* = 10	*n* = 11	*n* = 10	*n* = 8	
**Plasma**					
Cys (µM)	18.2 ± 2.0 ^a^	17.1 ± 1.0 ^a^	13.2 ± 2.0 ^a,b^	9.1 ± 1.0 ^b^	0.002
CySS (µM)	40.4 ± 2.0	40.9 ± 3.0	43.7 ± 3.0	33.4 ± 2.0	0.06
GSH (µM)	26.4 ± 5.0 ^a^	17.2 ± 2.0	20.7 ± 2.0	10.5 ± 2.0 ^b^	0.005
GSSG (µM)	1.6 ± 0.3	1.3 ± 0.3.0	1.3 ± 0.2	0.7 ± 0.2	0.2
Total Cys (µM)	114.1 ± 4.0 ^a^	109.4 ± 8.0 ^a^	113.8 ± 3.0 ^a^	81.0 ± 5.0 ^b^	0.004
Total GSH (µM)	44.8 ± 7.0 ^a^	30.4 ± 3.0 ^a,b^	36.5 ± 4.0 ^a^	17.0 ± 2.0 ^b^	0.001
**Liver**					
Cys (µM)	9.9 ± 2.0	7.2 ± 2.0	17.1 ± 9.0	11.8 ± 2.0	0.5
CySS (µM)	38.3 ± 7.0 ^a^	34.6 ± 6.0 ^a,b^	41.7 ± 8.0 ^a^	10.9 ± 2.0 ^b^	0.01
GSH (µM)	1884 ± 149.0 ^a^	1642 ± 137.0 ^a^	1432 ± 177 ^a,b^	982 ± 63 ^b^	0.002
GSSG (µM)	81.9 ± 13.0 ^a^	52.3 ± 5.0 ^a,b^	40.8 ± 7.0 ^b^	40.5 ± 4.0 ^b^	0.004
Total Cys (µM)	126.1 ± 15.0 ^a^	104.5 ± 14.0 ^a,b^	116.6 ± 18.0 ^a^	57.4 ± 5.0 ^b^	0.01
Total GSH (µM)	2087.2 ± 144 ^a^	1774.5 ± 142 ^a^	1530 ± 202 ^a,b^	1087 ± 66 ^b^	0.001

^a,b^ Different superscripted letters indicate statistically significant (*p* < 0.05) difference between groups; ^#^ Data expressed as mean ± SEM; ^†^ ANOVA.

### 3.4. Plasma Oxidative Stress Indices

**Figure 3:**
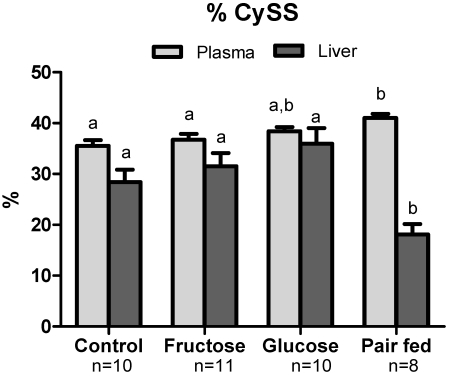
% CySS in plasma and liver. CySS was measured as percent of total Cys pool (Cys, CySS and Cys-GSH). Different letters (a,b) indicate statistically significant differences among respective groups. In the pair-fed group, plasma % Cyss was significantly increased while hepatic % Cyss was decreased, indicating oxidation of plasma but not liver.

**Figure 4:**
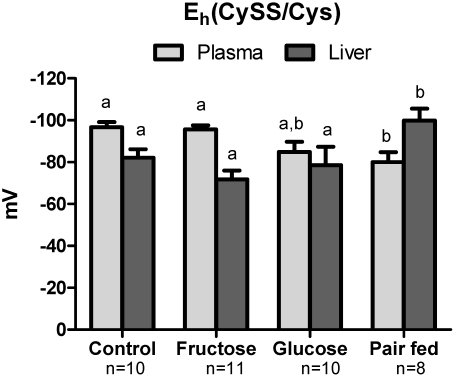
Cys/CySS redox potential (E_h_CySS) in plasma and liver. E_h_CySS is calculated using Nernst equation and expressed in mV. Different letters (a,b) indicate statistically significant difference among respective groups. In the pair fed group, the E_h_CySS was oxidized in plasma, while reduced in liver compared to other groups, indicating differential plasma but not hepatic oxidative stress.

Even with the decreased sulfur amino acid intake due to fructose and glucose, plasma Cys, total Cys (Cys + CySS + Cys-GSH) and total GSH concentration (GSH + GSSG + Cys-GSH) were similar to control mice (Table 2). In contrast, GSH level was lower in fructose-fed and glucose-fed mice compared to control. Interestingly, the fructose and pair-fed groups had the same sulfur amino acid intake, but the pair-fed animals had lower plasma Cys, total Cys, GSH and total GSH. Pair-fed animals also had an increased % CySS (Figure 3) indicating greater systemic oxidative stress. GSH/GSSG ratio, Cys/CySS ratio and % GSSG were not different across groups (data not shown). Plasma E_h_CySS was more oxidized (more positive E_h_) in pair-fed mice compared to control and fructose groups (Figure 4). E_h_GSSG values did not show significant differences between groups. 

### 3.5. Hepatic Oxidative Stress

**Figure 5:**
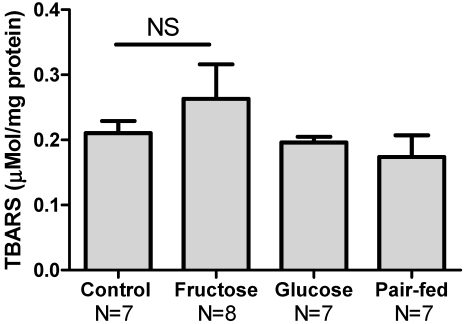
Results of thiobarbituric acid reactive substances (TBARS) assays shows that fructose and glucose feeding do not result in significant increases in hepatic lipid peroxidation. Data was analyzed via one-way ANOVA (*p* = 0.417).

Similar to data from the plasma, hepatic Cys and GSH content was not different in the control, fructose and glucose groups, but was decreased in the pair-fed animals (Table 2). Thiobarbituric acid reactive substances (TBARS) in the liver, a common metric of oxidative stress and lipid peroxidation, showed that TBARS was increased due to fructose treatment but this was not statistically significant (Figure 5). Importantly, the pair-fed group did not show evidence of increase. In contrast to the plasma data described above, the relative concentrations of CySS and Cys shifted towards a more reduced state (Figure 4). GSH/GSSG ratio, Cys/CySS ratio and % GSSG did not differ across the groups (data not shown).

In contrast to plasma, hepatic % CySS was lower and E_h_CySS was more reduced in the pair-fed group (Figure 3). E_h_GSSG in control, fructose, glucose and pair-fed groups were not significantly different. Thus, even though there is an indication of systemic oxidative stress in plasma, the results do not provide evidence for hepatic oxidative stress as measured by the tissue TBARS, Cys/CySS and GSH/GSSG pools. 

### 3.6. Antioxidant Protein Expression

**Figure 6:**
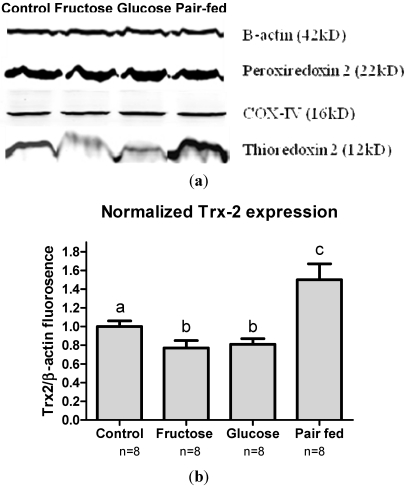
(**a**) Western blots of mitochondrial (Trx2) and cytoplasmic (Prx2) redox proteins in the liver in different study groups. Equal amounts of protein were loaded (20 µg) in each well as indicated by similar β-actin abundance. Similar abundance of COX-IV (mitochondrial control) indicated equal loading of mitochondrial protein. Similar Prx2 abundance signifies no oxidative stress in cytoplasm. Trx2 abundance was decreased in fructose and glucose but increased in pair fed group; (**b**) When normalized to β-actin, Trx2 abundance decreased in fructose (77%) and glucose (80%) groups while it increased in pair fed (150%) group compared to control (100%). This indicates mitochondrial predisposition to oxidative stress in fructose and glucose groups. Different letters (a,b,c) indicate statistically significant difference among respective groups.

Although the measurement of liver Cys/CySS and GSH/GSSG pools do not provide evidence of oxidative stress in the pair-fed group, these measures do not discriminate between effects in different subcellular compartments. Because oxidative stress can cause specific responses in subcellular compartments, we assessed the compartmental effects of fructose and glucose by measuring the abundance of mitochondrial Trx2 and cytoplasmic/nuclear Prx2. The abundance of these key compartment-specific antioxidant proteins was assessed using Western blotting. When normalized to β-actin (Figure 6a), Trx2 abundance was significantly lower in the fructose-fed and glucose-fed groups (23% and 20% lower) respectively, compared to control (Figure 6b). In contrast to that of the carbohydrate fed mice, the pair-fed group had significantly higher (150%) abundance of hepatic Trx2 compared to control. No difference was observed for the cytoplasmic antioxidant Prx2. Measurement of a component of the mitochondrial respiratory apparatus, cytochrome *c* oxidase IV (COX-IV) was used as a control for mitochondrial abundance, and this showed that the content of mitochondria did not differ among the groups.

## 4. Discussion

In the present study, we used a controlled, pair-feeding protocol to test the hypothesis that decreased sulfur amino acid intake contributes to oxidative stress in an fructose-sweetened beverage model of fatty liver. Both fructose and glucose-fed mice developed fatty liver, but animals pair-fed with only sulfur amino acid deficiency did not. Fructose-fed and glucose-fed animals had similar total energy intake compared to control and displayed a similar decrease in hepatic Trx2 abundance when compared to controls. Pair-fed animals had similar sulfur amino acid intake as the fructose-fed mice, and showed increased Trx2 abundance in liver. Together, the results show that sulfur amino acid insufficiency is not the cause of oxidative stress in the models of fructose-sweetened liquid-induced fatty liver. Instead, the results suggest that disproportionately high energy intake from simple sugar (48% and 63% of total energy intake in fructose-fed and glucose-fed mice) may predispose mitochondria to oxidative stress. The carbohydrate-mediated decrease in Trx2 abundance is potentially important in terms of cell viability and liver damage, because Trx2 protects against oxidant-mediated apoptosis and can regulate the mitochondrial permeability transition [56,57].

Fructose-sweetened beverage intake is associated with decreased solid food and protein ingestion in obese and normal weight human subjects [34]. Raben *et al.* [35] showed a significant decrease in protein intake after consumption of sucrose-containing liquid. In rodents, Jurgens *et al.* [45] showed that after *ad libitum* fructose-containing liquid consumption, solid food intake was decreased, but total energy consumption was the same as controls. Our data are consistent with these previous studies showing that fructose-sweetened liquid consumption is associated with decreased solid food consumption, thus leading to insufficient sulfur amino acid intake. Deficiency of methionine, an essential amino acid, has been implicated in alcoholic liver disease and liver cirrhosis [10], secondary to its important role as a precursor of GSH. However, the potential role of dietary methionine in the pathogenesis of fatty liver does not appear to have been previously studied.

Our results on hepatic TG content are similar to those of Ngo Sock *et al.* [58], who recently showed that hepatic TG content was similar in glucose and fructose-fed rodents. Several metabolic effects of high-fructose diet such as obesity, fatty liver, and insulin resistance can also be observed with high-glucose diet [32,58,59,60,61]. Also, in the long term, the effects of high fructose and high glucose may not be different. As fructose-sweetened beverage consumption is associated with high energy intake [58,59,62], it is unclear whether these hyperenergetic conditions can account for the metabolic effects of fructose [58,59]. 

Most of the studies on fructose-sweetened liquid and fatty liver use products of lipid peroxidation as markers of oxidative stress and only few studies have evaluated enzymatic antioxidant systems [32,63]. To our knowledge, this is the first study evaluating hepatic mitochondrial and cytoplasmic antioxidant proteins and calculating the redox potentials of major thiol-disulfide couples in fructose-sweetened liquid model of fatty liver. Sumida *et al.* [64] showed a correlation between serum Trx levels and hepatic fat content in patients with fatty liver and proposed its usefulness in discriminating fatty liver from NASH. That study did not, however, evaluate hepatic Trx levels or specific Trx isoenzymes. Our study measured Trx2 abundance, which was decreased due to fructose and glucose feeding. These results suggest a predisposition to mitochondrial oxidative stress, which could be a pathogenic mechanism of fatty liver. This is different from a rodent study by Mellor *et al.* [25], which did not reveal any difference in myocardial Trx2 gene abundance due to a high fructose (60%) diet. This discrepancy could be explained by metabolic differences in these two organs as fructose is primarily metabolized in liver. 

Machado *et al.* [63] showed that in patients with fatty liver, GSH metabolism is impaired towards oxidation, but plasma antioxidant markers do not correlate with hepatic redox states. We measured plasma redox potentials along with liver redox potentials to assess if there was any correlation between these two compartments. In our pair-fed group, hepatic E_h_CySS was reduced and Trx2 abundance increased; however plasma E_h_CySS was oxidized compared to control. These differences indicate that the redox compartments may act independently of each other [46]. Opposite redox effects in plasma and liver may be explained by energy restriction (40% of control in our study) leading to decreased metabolism in liver, which may result in less reactive oxygen species (ROS) formation in mitochondria. Alternatively, energy restriction can induce mitohormesis; an increased formation of ROS within mitochondria that causes an adaptive response providing stress resistance with a long-term reduction of oxidative stress [65]. Lastly, with the pair-fed animals being protein and energy restricted, they exhibited lower sulfur amino acid levels in both plasma and the liver. This can be explained by a marked reduction in protein synthesis and increase in protein half-lives; therefore, a lack of protein intake and decreased protein turnover could result in lower levels of tissue and circulating amino acids. 

When comparing fructose-fed animals to glucose-fed animals, both groups had similar sulfur amino acid intake, total caloric intake, plasma and liver redox states and decrease in hepatic Trx2 abundance. A predisposition to hepatic mitochondrial oxidative stress as suggested by decreased Trx2 may be due to disproportionate energy intake from simple sugars, rather than decreased sulfur amino acid intake. 

One of the limitations of our study was the design of the pair-fed group. We matched the fructose group for solid food intake but not for total energy intake. Because the restricted diet intake in the pair-fed animals also reduced caloric intake, a more precise design for the sulfur amino acid effect would be to supplement the diet composition of the pair-fed control group with non-carbohydrate energy sources (e.g., lipid) such that caloric intake in all four groups was similar. Future experiments with matched pair feeding according to macronutrient composition could potentially improve the models for fructose-sweetened liquid-induced fatty liver. 

## 5. Conclusion

Fructose-sweetened liquid predisposes the liver to mitochondrial oxidative stress and causes fatty liver without plasma or cytoplasmic oxidative stress. The hypocaloric, pair-fed group displayed plasma, but not hepatic oxidative stress or fatty liver. Therefore, decreased sulfur amino acid intake, as occurs in models commonly used to study fructose-induced fatty liver, is not a cause of hepatic oxidative stress in this model and does not provide an explanation for fructose-induced lipid accumulation in this model. 

## Implications

The study rules out a potential artifact of an important model used to study fructose-induced fatty liver. The study additionally shows the potential importance of mitochondrial oxidative stress in pathogenesis of fructose-induced fatty liver and that fructose and glucose-sweetened beverages may have similar hepatic metabolic, as evidenced by the equivalent liver TG outcomes, and redox effects.
